# Association between Bar Closing Time, Alcohol Use Disorders and Blood Alcohol Concentration: A Cross-Sectional Observational Study of Nightlife-Goers in Perth, Australia

**DOI:** 10.3390/ijerph19127026

**Published:** 2022-06-08

**Authors:** William Gilmore, Martyn Symons, Wenbin Liang, Kathryn Graham, Kypros Kypri, Peter Miller, Tanya Chikritzhs

**Affiliations:** 1 National Drug Research Institute and enAble Institute, Faculty of Health Sciences, Curtin University, Perth, WA 6845, Australia; martyn.symons@curtin.edu.au (M.S.); t.n.chikritzhs@curtin.edu.au (T.C.); 2 School of Public Health, Fujian Medical University, Fuzhou 350108, China; wenbin_liang@fjmu.edu.cn; 3 Institute for Mental Health Policy Research, Centre for Addiction and Mental Health, Toronto, ON M5S 2S1, Canada; kgraham@uwo.ca; 4 School of Medicine and Public Health, University of Newcastle, Callaghan, NSW 2308, Australia; kypros.kypri@newcastle.edu.au; 5 Centre for Drug Use, Addictive and Anti-Social Behaviour Research, Deakin University, Geelong, VIC 3220, Australia; peter.miller@deakin.edu.au

**Keywords:** nightlife-goers, bars, on-trade licensed outlets, alcohol use disorders, AUDIT-C, blood alcohol concentration, BAC, trading hours, closing times, alcohol policy

## Abstract

Introduction and aims: Associations between bar trading hours, a government lever for controlling alcohol availability, nightlife-goer intoxication levels and their likelihood of alcohol use disorder (AUD) have not been explored. We investigated whether: (i) participant AUD was associated with blood alcohol concentration (BAC); and, (ii) any association between AUD and BAC was moderated by participant preferred bar (i.e., venue spent most time at) closing time. Design and methods: A cross-sectional observational study using a sample of nightlife-goers who went out drinking in Perth, Western Australia, on weekends in 2015-16. Participants who reported alcohol use that night and spent most time in a bar (n = 667) completed street intercept surveys including AUDIT-C (n = 459) and provided a breath sample to estimate BAC (n = 651). We used gender-specific multinomial logistic regression models to explore associations between participant AUDIT-C score (1–4, lower risk; 5–7, hazardous; 8–12, active AUD), preferred bar type (standard vs. late closing time based on absence or presence of an extended trading permit) and BAC (male: 0–0.049, 0.05–0.099, ≥0.1 g/100 mL; female: 0–0.049, 0.05–0.079, ≥0.08 g/100 mL). Results: Males with active AUD (RR = 3.31; 95% CI 1.30–8.42; *p* = 0.01) and females with hazardous/active AUD (RR = 9.75; 95% CI 2.78–34.21; *p* < 0.001) were both more likely to have high-range BAC than their counterparts typically drinking at lower risk. We also found preferred bar type moderated the association between AUDIT-C score and BAC for some males but no females. Males with active AUD and high-range BAC were less likely to prefer late closing bars than males usually drinking at lower risk and high-range BAC (RR = 0.12; 95% CI 0.02–0.96; *p* = 0.046). Discussion and conclusions: Our study provides evidence of positive associations between AUD and acute intoxication among nightlife-goers and on the moderating effect of bar closing times among males.

## 1. Introduction

Availability theory proposes that increased alcohol availability in a community will increase alcohol consumption and both short-term and long-term alcohol-related harms, and the distribution of harms will vary according to differing drinking patterns [1]. Stipulating the days and hours that alcohol outlets can trade, via a liquor licensing system, is one government lever for controlling alcohol availability in a community. Systematic reviews [2,3,4,5,6,7,8] and meta-analyses [9] of international research evaluating both community-wide restrictions and extensions to alcohol outlet trading hours have concluded that community-level consumption and related harm are positively associated with outlet trading hours. Associations between bar trading hours, nightlife-goer intoxication levels and their likelihood of alcohol use disorder (AUD) have not been explored.

It might be expected that people with AUD would have high blood alcohol concentrations (BAC) when drinking. Studies of the association between AUD and BAC, to the best of our knowledge, are limited to trauma patients who had BAC calculated from venous blood on presentation and subsequently completed an AUD screen of their usual drinking patterns. Although not tending to be the main focus of these studies, one prospective cohort study of patients admitted to a US trauma centre found evidence of a moderate positive association between AUD and BAC (Spearman’s ρ = 0.45) [10]. Another, a US retrospective study of admitted intensive care unit trauma patients also found evidence of a positive association between AUD and BAC (Kruskal-Wallis *p <* 0.001) [11].

Research evidence for an association between AUD and outlet trading hours is scant. This is of interest as one might expect that drinkers with AUD may gravitate towards outlets with longer trading hours due to increased alcohol availability. A German study on liquor store trading restrictions between 10 p.m. and 5 a.m. found evidence of positive associations with hospitalisations for mental and behavioural disorders due to alcohol use by younger males (8% reduction) and females (4% reduction) [12]. Studies from Perth, Australia, have found evidence of positive associations between likelihood of AUD and bar trading hours. Based on self-reported past week consumption, a study of male drinkers found those who drank at bars opening at 6 a.m. or 7 a.m. were more likely to have AUD compared to males who drank at bars opening later at 10 a.m. (47% vs. 37%) [13]. A recent nightlife study using the same survey data as the current study found evidence that, based on self-report of past year consumption using AUDIT-C (3 question short form of AUDIT), females drinking hazardously chose to spend most time drinking at ‘late’ closing bars compared to bars closing at midnight (Friday, Saturday) or 10 p.m. (Sunday) (OR = 3.48; 95% CI 1.47–8.23; *p* = 0.01) [14]. There was no evidence of association for males.

Evidence regarding the association between outlet trading hours and BAC is also scant. An evaluation of restrictions that imposed 3 a.m. alcohol sale cessation across a nightlife area of Brisbane, Australia, found fewer highly intoxicated (≥0.1 g/100 mL BAC) versus moderately intoxicated (0.050–0.099 g/100 mL BAC) nightlife-goers in the month following the restriction compared to the month before (RR = 0.58; 95% CI = 0.43, 0.79) [15]. These findings persisted despite a loophole allowing some premises to trade until 5 a.m. Nightlife research using street intercept survey methodology that includes breathalysing nightlife goers spans North America [16], Europe [17] and Australasia [18]. These studies generally find that average patron BAC increases through the night [19], however, few (if any) have also reported on ‘usual’ drinking patterns, or likelihood of AUD among participants.

Gender differences in alcohol consumption and experienced harms have been shown to exist in national surveys of nightlife-goers and the general population and in analyses of health data [18,20,21]. Despite a narrowing gap between genders over time, with women catching up with men in their alcohol consumption, it is still men who, on average, consume the most alcohol, have riskier patterns of consumption [20] and who are overrepresented in harm statistics [21]. Alcohol availability studies are therefore enhanced when analyses are able to distinguish by gender [14].

To our knowledge, this is the first study to link BACs and usual drinking patterns of nightlife-goers to the trading hours of the bar they chose to spend most time at on their night out (i.e., their preferred bar). As bar trading hours are the potentially modifiable environmental factor among these variables, this study will be of importance in future government decisions regarding bar trading hour regulations. Using a sample of nightlife-goers who went out drinking in Perth, Western Australia, we aimed to investigate by gender whether: (i) participant likelihood of AUD, based on self-reported past year alcohol use, was associated with BAC, an objective measure of alcohol intoxication; and, (ii) closing time (standard vs. late) of participant preferred bar moderated (i.e., influenced the strength and/or direction of association [22]) any association between participant AUD and BAC. We hypothesised that: (i) participants with a usual drinking pattern indicating hazardous use or active AUD would be more likely to have a high-range BAC (≥0.1 g/100 mL) on the night of survey compared to typically lower risk drinkers regardless of preferred bar type (standard vs late closing time) (Aim 1); (ii) within categories of AUD risk (lower risk, hazardous, active AUD), participants with a high-range BAC would prefer late closing bars to standard closing bars (Aim 2); and, (iii) gender differences would occur across these associations.

## 2. Methods

### 2.1. Street Intercept Surveys

Trained field workers undertook street intercept surveys between November 2015 and April 2016 in metropolitan Perth’s major nightlife precincts (Perth City; Northbridge; Leederville; Fremantle). To approximate a random sample, field workers invited every third person in public spaces to participate (8 p.m. to 3 a.m. Friday and Saturday; 8 p.m. to midnight Sunday). We achieved a response rate of 89%, not including passers-by who did not engage with field workers to hear the purpose of the survey. Sample size quotas of 200 by gender and preferred bar type were set. The street intercept approach in this field is well established [14,17,23] and is successful in recruiting samples of nightlife-goers [16].

Following participants’ informed consent, field workers entered survey responses on their smartphones in Tap Forms™. Participants self-reported gender, birth year and usual occupation while the survey app captured date and time automatically. Participants answered the three AUDIT-C questions assessing: (i) frequency of drinking; (ii) typical number of drinks consumed on a drinking occasion; and, (iii) frequency of six or more standard drinks, all over the past year [24]. AUDIT-C is a quick, simple, reliable (Cronbach’s alpha = 0.7 on another Australian sample in a non-clinical setting [25]) and well validated tool to screen for hazardous drinking or active AUD based on past year drinking pattern [25,26,27,28,29]. Participants provided a breath sample through a calibrated Andatech^®^ AlcoSense^®^ Prodigy Fuel Cell Breathalyser to estimate their BAC (calibration date: 10 September 2015; accuracy: ±0.005 at 0.1 g/100 mL).

The Western Australian liquor licensing system allows bars to apply for extended trading hour permits [30]. Standard closing for bars in 2015 was midnight Monday to Saturday and 10 p.m. Sunday. At the time of study initiation granted permits allowed bars to trade up until 2 a.m. or 3 a.m. Monday to Saturday and until midnight Sunday (i.e., late closing). If participants responded yes to drinking alcohol at one or more licensed venues, field workers asked for venue names and an estimate of how much time was spent at each in order to establish the bar at which they had chosen to spend most time that night. We ceased Sunday field work at the end of 2015 after two nights of surveys because legislation came in to effect relaxing bar trading hours (midnight became ‘standard’ Sunday closing time) [14,31].

Participants answered other questions related to their drinking behaviours that night including: Had they drunk any alcohol that night (Y/N)? How long had they been drinking (Hours)? Had they been drinking at licensed venues (Y/N)? Had they been pre-drinking (Y/N)? Had they drunk energy drinks (Y/N)? Was it a typical night out for them (Yes; No, smaller than usual; No, bigger than usual)?

### 2.2. Survey Data

We categorised AUDIT-C scores into three groups using the same raw score cut-offs for males and females: 1–4, lower risk drinker; 5–7, hazardous drinker; 8–12, drinker with active AUD [32]. We further categorised females into two groups due to small numbers in the higher risk categories (5–12, hazardous/active AUD). As male and female BAC distributions were positively skewed, ruling out linear regression, we categorised them. We grouped BAC for males into three levels of intoxication: 0–0.049 g/100 mL; 0.05–0.099 g/100 mL; ≥0.1 g/100 mL, with 0.05 g/100 mL being the drink drive limit in Australia at which a person is deemed legally intoxicated. We grouped BAC for females with lower thresholds due to the different data distribution from males and as females are typically affected by alcohol at a lower BAC than males [33]: 0–0.049 g/100 mL; 0.05–0.079 g/100 mL; ≥0.08 g/100 mL. Records with BAC readings exceeding 0.35 g/100 mL were excluded as erroneous (n = 5) [34]. As it is typical for nightlife-goers to drink at a number of different venues on a night out (e.g., restaurant, bar, nightclub), we used venue names to distinguish venues (i.e., bar vs. other) and the closing time of each bar (standard vs. late) using Department of Local Government Sport and Cultural Industries extended trading permit records and bar websites. We then used venue where most time was spent to define participant ‘preferred bar type’ and assumed this is where they consumed most alcohol.

We calculated participant age using date of survey and year of birth then categorised into four approximately equal groups based on the distribution of the data: 18–21; 22–25; 26–29; ≥30. We classified occupation according to the Australian and New Zealand Standard Classification of Occupations (plus an ‘Other’ category to capture students, stay-at-home parents, unemployed) [35] and grouped as follows: manager/professional; technician/trade/labourer; community/personal service; clerical/administrative/sales; other. We dichotomised time of survey into ‘before midnight’ and ‘midnight and after’, reflecting the distinction between standard and late closing bars. In order to reflect typical night-time drinking occasions, we categorised day of survey (i.e., Friday, Saturday or Sunday) according to when data collection sessions were initiated, e.g., surveys undertaken between 10 p.m. Friday night and 2 a.m. the following morning were all considered a ‘Friday’ night survey.

### 2.3. Statistical Analysis

We used Pearson’s chi-square tests and one-way analysis of variance to explore gender-specific bivariate associations between preferred bar type and AUDIT-C score, age, occupation, day of survey, time of survey, drinking session duration, whether it was a typical night out, pre-drinking and energy drink use. We used multinomial logistic regression models with backward stepwise selection approach to investigate associations between AUDIT-C score and BAC and adjusted for the range of potential confounders listed above. We ran six initial gender-specific models to explore the overall association between AUDIT-C score and BAC and the associations by preferred bar type. We then ran two gender-specific models with preferred bar type as an interaction term to determine whether preferred bar type moderated any association between AUDIT-C score and BAC. Likelihood ratio χ^2^ tests assessed model goodness-of-fit. We used IBM SPSS Statistics v27.0 (IBM Corp, Armonk, NY, USA) for all analyses [36].

### 2.4. Ethics

We conducted this study in accordance with the National Statement on Ethical Conduct in Human Research and received ethics approval from Curtin University’s Human Research Ethics Committee (HR154/2015). Participants provided informed consent to field workers who recorded responses in a smartphone survey app.

## 3. Results

Of the 667 participants (males n = 454, females n = 213) who had been drinking and preferred a bar to other venue (e.g., nightclubs), 651 provided a valid BAC, 459 completed the AUDIT-C, 289 preferred standard closing bars and 378 preferred late closing bars (Table 1). Around one-third of male and female participants returned BAC readings of ≥0.1 g/100 mL or ≥0.08 g/100 mL, respectively, regardless of their preference for standard or late closing bars. A large proportion of participants were either typically hazardous drinkers or had active AUD (83% males, 65% females).

Gender-specific bivariate analyses indicated evidence of a positive association between female AUDIT-C score and preferred bar type. There was no evidence of association between BAC and preferred bar type for either gender. Of the other participant characteristics, female preferred bar type was positively associated with age and pre-drinking. Male preferred bar type was positively associated with energy drink use. Of the survey characteristics, weekday was positively associated with both male and female preferred bar type, with a higher proportion of Friday night participants preferring late closing bars for both genders. Time of day was positively associated with male preferred bar type but not female. For the following multinomial logistic regression model results, likelihood ratio χ^2^ tests gave no cause for concern regarding model goodness-of-fit (Table 2 and Table 3).

### 3.1. AUDIT-C Score and BAC by Preferred Bar Type

**Figure 1:**
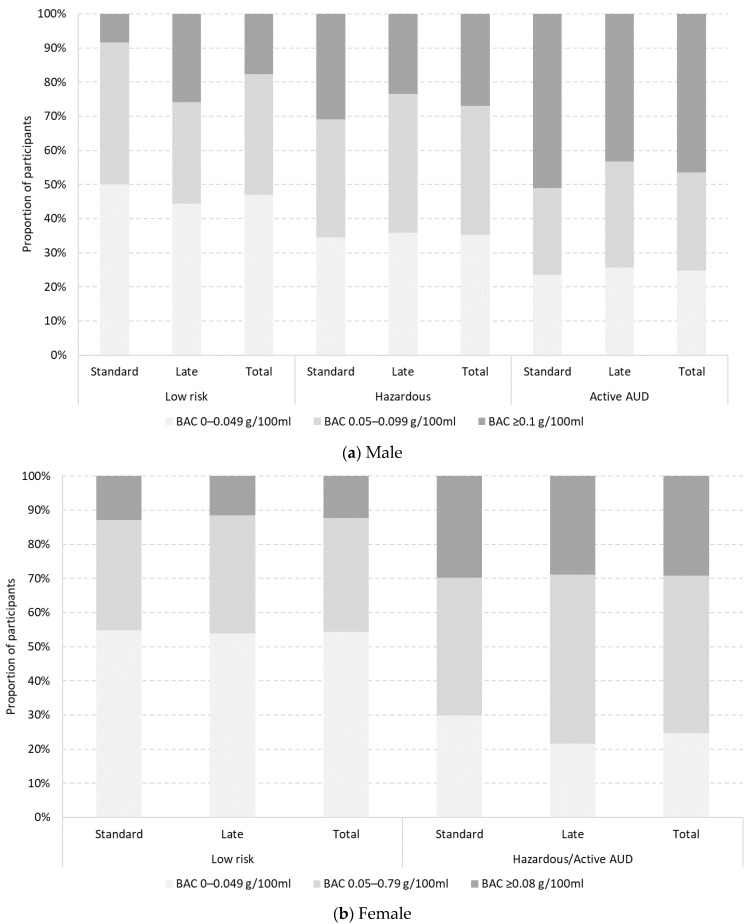
Proportion of male and female participants according to BAC (g/100 mL) by AUDIT-C score (lower risk, hazardous, active AUD) and preferred bar type (standard vs. late).

Overall, males with active AUD (RR = 3.31; 95% CI 1.30–8.42; *p* = 0.01) and females with hazardous/active AUD (RR = 9.75; 95% CI 2.78–34.21; *p <* 0.001) were more likely to have a high-range BAC than lower risk drinkers (Table 2 and Figure 1). When stratifying by preferred bar type, associations held among males (RR = 13.42; 95% CI 2.47–72.97; *p* = 0.003) and females (RR = 6.18; 95% CI 1.35–28.21; *p* = 0.02) preferring standard closing bars and among females preferring late closing bars (RR = 21.89; 95% CI 3.50–137.10; *p <* 0.001) (Table 2 and Figure 1). For males, high-range BAC was negatively associated with not having pre-drunk when not accounting for preferred bar type but positively associated with usually having a smaller night out among those preferring late closing bars and with drinking session duration regardless of preferred bar type. For females preferring late closing bars, high-range BAC was positively associated with drinking session duration and being surveyed after midnight and negatively associated with younger age groups (18–21; 22–25).

### 3.2. AUDIT-C Score and Preferred Bar Type on BAC

When preferred bar type was included in gender-specific models as an interaction term (Table 3 and Figure 1), there was evidence of association between AUDIT-C score, preferred bar type and BAC for some males but no females. Males with active AUD and a high-range BAC were less likely to prefer late closing bars to standard closing bars than males drinking at lower risk with a high-range BAC (RR = 0.12; 95% CI 0.02–0.96; *p* = 0.046). For males, high-range BAC was positively associated with drinking session duration and mid-range BAC was positively associated with pre-drinking and drinking session duration. For females, high-range BAC was positively associated with drinking session duration and being surveyed after midnight and negatively associated with both technical and clerical occupations and mid-range BAC was positively associated with usually having a smaller night out and negatively associated with a clerical occupation.

## 4. Discussion

In nightlife areas of Perth, male bar patrons with active AUD were around three times as likely to have a BAC reading exceeding 0.099 g/100 mL than males usually drinking at lower risk. Females with usual drinking patterns indicative of hazardous use or active AUD were around ten times as likely to have a BAC exceeding 0.079 g/100 mL than their lower risk drinking counterparts. These findings that increased risk of AUD was associated with increased BAC among nightlife-goers (when not adjusting for the closing times of their preferred bars) are as we expected. This is the first nightlife study to have explored this association, but there is evidence among trauma patients that those with higher likelihood of AUD will have a higher BAC on presentation [10,11].

After differentiating participants according to their preferred bar type, we found there was a strong positive association between AUDIT-C score and BAC for males from standard closing bars but no evidence of association for males from late closing bars. For females, there was evidence of a strong positive association between AUDIT-C score and BAC for females from late closing and standard closing bars. We had expected that regardless of preferred bar type, participants typically drinking at hazardous levels or with AUD would be more likely to have a high-range BAC on a night out. In terms of the gender differences, we found by preferred bar type, it is important to note that venues across and within each bar type (standard vs. late), despite having certain similarities in how they function by virtue of their liquor licensing classification, may differ from each other in many ways. A wide range of contextual factors (e.g., bar size, live entertainment, dancefloor, drink promotions, entry and serving practices) may influence what clientele a bar attracts. These are potential confounders that we were unable to adjust for, but collection of such contextual information should be considered in future studies.

We found preferred bar type moderated the association between AUDIT-C score and BAC for some males but no females. Males with active AUD with a high-range BAC on their night out were less likely to prefer late closing bars to standard closing bars than males usually drinking at lower risk who had a high-range BAC. As late trading increases the hours of alcohol availability thus giving more opportunity for intoxication, we had expected that within categories of AUD risk, participants preferring late closing bars would be more likely to have a high-range BAC. Among male nightlife-goers drawn to late trading bars, it is those with typically lower risk drinking patterns who are more likely to reach BACs ≥ 0.1 g/100 mL than those with AUD. It may be that males with AUD are less influenced by trading hours when out drinking to intoxication compared to male lower risk drinkers who are on a big night out. Half of males reported that it was not a typical night out for them, and this may go part way to explaining the slightly unexpected findings. Regarding no evidence of association for females in the interaction model, as well as the lack of contextual differences between bars included in the models that may explain gender differences, sample size was approximately half that of males and this may have affected statistical power.

Despite an inclination towards relaxation of outlet trading hours by liquor licensing authorities globally, there is mounting evidence that it may lead to increased consumption and harm. In Western Australia, at least, there have been recent examples of bar trading hours easing on Sundays and there are plans for easing of Sunday liquor store restrictions in remote areas [31]. Extended trading hour permits for bars in Western Australia fall create a loophole in liquor licensing laws and provide bars with permits an exemption to the rule. This study provides new evidence of an association between outlet closing times and alcohol consumption that is of relevance to decision makers—male nightlife-goers, albeit typically lower risk drinkers, who are highly intoxicated when out drinking prefer late closing bars with extended trading hour permits.

### Limitations

When classifying participants as preferring standard vs. late closing bars, we assumed that time spent in a venue was positively associated with quantity of alcohol consumed. However, a participant classified as preferring a standard closing bar, for example, may have spent an hour and a half drinking two units of alcohol in a standard closing bar and one hour drinking one unit in each of three late closing bars. It is also important to note that half of participants reported not being on a typical night out, with around a quarter reporting usually having a bigger night out and a quarter usually having a smaller night out. We have only presented evidence of cross-sectional associations between nightlife-goers’ AUDIT-C score, the closing time of their preferred bar and their BAC not the directions of these associations. BAC was the only objective measure collected and as cognitive ability declines with alcohol intoxication [37] we must be cautious with measures collected via self-report. Finally, our findings may not be generalisable to nightlife areas in other cities.

## 5. Conclusions

Our study provides evidence of positive associations between alcohol use disorders and acute intoxication among nightlife-goers and on the moderating effect of bar closing times among males.

## Figures and Tables

**Table 1:** Gender-specific descriptive statistics and bivariate analyses for participant and survey characteristics by preferred bar type (standard vs. late).

Variables	Male							Female						
	Standard		Late		Total			Standard		Late		Total		
Participant Characteristics	n	%	n	%	n	%		n	%	n	%	n	%	
BAC (g/100 mL)							χ^2^(2) = 0.6, *p* = 0.75							χ^2^(2) = 3.3, *p* = 0.19
0–0.049	68	35	89	36	157	35		41	50	47	37	88	42	
0.05–0.079 (female)	-	-	-	-	-	-		15	18	28	22	43	21	
≥0.08 (female)	-	-	-	-	-	-		26	32	51	40	77	37	
0.05–0.099 (male)	59	30	82	33	141	32		-	-	-	-	-	-	
≥0.1 (male)	67	35	78	31	145	33		-	-	-	-	-	-	
Total	194	100	249	100	443	100		82	100	126	100	208	100	
AUDIT-C score							χ^2^(2) = 1.2, *p* = 0.54							χ^2^(1) = 5.7, *p* = 0.02
1–4 lower risk	27	19	27	16	54	17		29	46	23	27	52	35	
5–12 hazardous/active AUD (f)	-	-	-	-	-	-		34	54	62	73	96	65	
5–7 hazardous (m)	58	41	67	39	125	40		-	-	-	-	-	-	
8–12 active AUD (m)	55	39	77	45	132	42		-	-	-	-	-	-	
Total	140	100	171	100	311	100		63	100	85	100	148	100	
Age							χ^2^(3) = 7.0, *p* = 0.07							χ^2^(3) = 8.2, *p* = 0.04
18–21	24	12	46	18	70	15		19	22	39	31	58	27	
22–25	48	24	73	29	121	27		25	29	41	32	66	31	
26–29	59	29	56	22	115	25		24	28	16	13	40	19	
≥30	70	35	76	30	146	32		18	21	31	24	49	23	
Total	201	100	251	100	452	100		86	100	127	100	213	100	
Occupation							χ^2^(4) = 8.4, *p* = 0.08							χ^2^(4) = 3.9, *p* = 0.42
Manager/professional	65	33	88	36	153	35		6	7	8	7	14	7	
Technician/trade/labourer	15	8	18	7	33	7		12	14	25	20	37	18	
Community/personal service	7	4	24	10	31	7		22	26	28	23	50	24	
Clerical/administrative/sales	33	17	31	13	64	15		17	20	33	27	50	24	
Other	77	39	83	34	160	36		28	33	29	24	57	27	
Total	197	100	244	100	441	100		85	100	123	100	208	100	
Pre-drinking							χ^2^(1) = 3.8, *p* = 0.05							χ^2^(1) = 8.8, *p <* 0.01
No	108	53	110	44	218	48		53	62	52	41	105	49	
Yes	95	47	140	56	235	52		33	38	75	59	108	51	
Total	203	100	250	100	453	100		86	100	127	100	213	100	
Energy drink use							χ^2^(1) = 8.3, *p <* 0.01							χ^2^(1) = 1.4, *p* = 0.24
No	185	91	205	82	390	86		79	92	110	87	189	89	
Yes	18	9	46	18	64	14		7	8	17	13	24	11	
Total	203	100	251	100	454	100		86	100	127	100	213	100	
Typical night out? ^±^							χ^2^(2) = 2.0, *p* = 0.37							χ^2^(2) = 2.9, *p* = 0.24
No, usually smaller	32	25	44	33	76	29		16	27	20	25	36	26	
No, usually bigger	27	21	28	21	55	21		16	27	13	16	29	21	
Yes	68	54	62	46	130	50		27	46	46	58	73	53	
Total	127	100	134	100	261	100		59	100	79	100	138	100	
Session duration (Hours)	n	Mean (SD)	n	Mean (SD)	n	Mean (SD)		n	Mean (SD)	n	Mean (SD)	n	Mean (SD)	
	198	5.05 (2.52)	246	4.81 (2.73)	444	4.92 (2.64)	F(1, 442) = 0.9, *p* = 0.35	86	4.41 (2.02)	126	4.52 (2.27)	212	4.47 (2.17)	F(1, 210) = 0.1, *p* = 0.74
Survey characteristics	n	%	n	%	n	%		n	%	n	%	n	%	
Day							χ^2^(2) = 21.0, *p <* 0.001							χ^2^(2) = 10.1, *p <* 0.01
Friday	48	24	108	43	156	34		19	22	52	41	71	33	
Saturday	118	58	119	47	237	52		62	72	64	50	126	59	
Sunday	37	18	24	10	61	13		5	6	11	9	16	8	
Total	203	100	251	100	454	100		86	100	127	100	213	100	
Time							χ^2^(1) = 10.9, *p <* 0.001							χ^2^(1) = 3.6, *p* = 0.06
Before midnight	134	66	127	51	261	57		52	60	60	47	112	53	
Midnight and after	69	34	124	49	193	43		34	40	67	53	101	47	
Total	203	100	251	100	454	100		86	100	127	100	213	100	

f: Female. m: Male. n: Sample size. Not all % totals sum to 100 due to rounding. ^±^ Small or big night out are colloquialisms regarding level of perceived intoxication.

**Table 2:** Gender-specific multinomial logistic regression models: Association between participant AUDIT-C and BAC by preferred bar type (standard, late, total) adjusting for survey and participant characteristics ^±^.

Variables ^±^	Male										Female									
	BAC 0.05–0.099 g/100 mL					BAC ≥ 0.1 g/100 mL					BAC 0.05–0.079 g/100 mL					BAC ≥ 0.08 g/100 mL				
	n	RR	LCI	UCI	*p*-Value	n	RR	LCI	UCI	*p*-value	n	RR	LCI	UCI	*p*-Value	n	RR	LCI	UCI	*p*-Value
Standard closing time models	Likelihood ratio χ^2^(6) = 26.8, *p <* 0.001										Likelihood ratio χ^2^(6) = 13.6, *p* = 0.04									
AUDIT-C score																				
1–4 lower risk [Ref]	10					2					5					3				
5–12 haz/active AUD (f)	-	-	-	-	-	-	-	-	-	-	5	2.06	0.41	10.33	0.38	11	6.18	1.35	28.21	0.02
5–7 hazardous (m)	19	1.17	0.40	3.41	0.77	17	5.07	0.95	27.05	0.06	-	-	-	-	-	-	-	-	-	-
8–12 active AUD (m)	13	1.35	0.42	4.36	0.61	26	13.42	2.47	72.97	<0.01	-	-	-	-	-	-	-	-	-	-
Typical night out?																				
No, usually smaller											6	9.25	1.33	64.32	0.02	3	1.33	0.22	8.09	0.76
No, usually bigger											2	1.47	0.17	12.64	0.72	3	0.52	0.10	2.75	0.44
Yes											3					8				
Session duration	42	1.24	1.00	1.54	0.053	45	1.41	1.13	1.76	<0.01										
Late closing time models	Likelihood ratio χ^2^(14) = 43.0, *p <* 0.001											Likelihood ratio χ^2^(12) = 38.1, *p <* 0.001								
AUDIT-C score																				
1–4 lower risk [Ref]	5					5					6					3				
5–12 haz/active AUD (f)	-	-	-	-	-	-	-	-	-	-	17	3.22	0.79	13.12	0.10	24	21.89	3.50	137.10	<0.001
5–7 hazardous (m)	24	0.93	0.21	4.12	0.93	13	1.06	0.24	4.76	0.94	-	-	-	-	-	-	-	-	-	-
8–12 active AUD (m)	18	2.10	0.50	8.81	0.31	19	1.07	0.24	4.87	0.93	-	-	-	-	-	-	-	-	-	-
Age																				
18–21											9	0.56	0.09	3.61	0.54	4	0.03	0.003	0.29	<0.01
22–25											7	0.25	0.04	1.46	0.12	10	0.07	0.01	0.48	<0.01
26–29											1	0.32	0.02	5.36	0.43	3	0.36	0.03	4.54	0.43
≥30 [Ref]											6					10				
Pre-drinking																				
No	11	0.16	0.06	0.46	<0.001	14	0.39	0.13	1.13	0.08										
Yes [Ref]	36					23														
Typical night out?																				
No, usually smaller	17	2.14	0.67	6.90	0.20	17	3.92	1.16	13.22	0.03										
No, usually bigger	8	0.86	0.26	2.91	0.81	8	1.28	0.35	4.67	0.71										
Yes	22					12														
Session duration	47	1.17	0.89	1.54	0.26	37	1.50	1.14	1.98	<0.01	23	1.48	1.04	2.10	0.03	27	1.64	1.12	2.38	0.01
Time																				
Before midnight [Ref]	20					18					14					11				
Midnight and after	27	3.07	1.15	8.23	0.03	19	1.86	.65	5.31	0.24	9	1.21	0.32	4.50	0.78	16	7.26	1.58	33.31	0.01
Total models	Likelihood ratio χ^2^(8) = 55.6, *p <* 0.001											Likelihood ratio χ^2^(18) = 51.0, *p <* 0.001								
AUDIT-C score																				
1–4 lower risk [Ref]	18					9					10					5				
5–12 haz/active AUD (f)	-	-	-	-	-	-	-	-	-	-	21	3.26	1.09	9.73	0.03	32	9.75	2.78	34.21	<0.001
5–7 hazardous (m)	45	1.31	0.61	2.80	0.49	32	1.76	0.70	4.47	0.23	-	-	-	-	-	-	-	-	-	-
8–12 active AUD (m)	36	1.12	0.50	2.54	0.78	58	3.31	1.30	8.42	0.01	-	-	-	-	-	-	-	-	-	-
Occupation																				
Manager/professional											1	0.25	0.02	3.97	0.33	3	0.47	0.05	4.87	0.53
Technician/trade/labourer											8	0.32	0.06	1.67	0.18	7	0.11	0.02	0.64	0.01
Community/personal service											7	0.52	0.12	2.22	0.52	8	0.39	0.09	1.80	0.23
Clerical/administrative/sales											6	0.13	0.03	0.62	0.13	6	0.07	0.01	0.37	<0.01
Other [Ref]											9					13				
Pre-drinking																				
No	38	0.40	0.22	0.73	<0.01	39	0.52	0.28	0.97	0.04										
Yes [Ref]	61					60														
Typical night out?																				
No, usually smaller											11	3.71	1.05	13.04	0.04	10	2.23	0.56	8.85	0.25
No, usually bigger											5	0.59	0.16	2.13	0.42	7	0.55	0.15	2.05	0.38
Yes											15					20				
Session duration	99	1.21	1.04	1.40	0.01	99	1.43	1.23	1.67	<0.001	31	1.21	0.90	1.61	0.21	37	1.50	1.10	2.03	<0.01
Time																				
Before midnight [Ref]											18					18				
Midnight and after											19	2.58	0.83	8.06	0.10	13	4.46	1.36	14.62	0.01

f: Female. m: Male. n: Sample size. RR: Risk ratio. L/UCI: 95% lower/upper confidence interval. [Ref]: Reference group. **^±^** energy drink use and weekday were non-contributing variables in all models, whether it was a typical night out was a non-contributing variable in the male standard model, drinking session duration was a non-contributing variable in the female standard model, age and whether it was a typical night out were non-contributing variables in the male late model, pre-drinking was a non-contributing variable in the female late model, occupation, whether it was a typical night out and time of survey were non-contributing variables in the male combined model, pre-drinking was a non-contributing variable in the female combined model. These non-contributing variables were removed in the backward stepwise selection approach.

**Table 3:** Gender-specific multinomial logistic regression models: Two-way interaction effect between AUDIT-C and preferred bar type (standard vs. late) on BAC adjusting for survey and participant characteristics ^±^.

Variables ^±^	Male										Female									
	BAC 0.05–0.099 g/100 mL					BAC ≥0.1 g/100 mL					BAC 0.05–0.079 g/100 mL					BAC ≥0.08 g/100 mL				
	n	RR	LCI	UCI	*p*-Value	n	RR	LCI	UCI	*p*-Value	n	RR	LCI	UCI	*p*-Value	n	RR	LCI	UCI	*p*-Value
AUDIT-C by preferred bar type																				
5–12 × Late (f)	-	-	-	-	-	-	-	-	-	-	16	2.26	0.24	21.09	0.48	21	2.45	0.19	31.86	0.49
5–7 × Late (m)	26	1.29	0.28	5.96	0.74	15	0.18	0.02	1.40	0.10	-	-	-	-	-	-	-	-	-	-
8–12 × Late (m)	23	1.06	0.21	5.41	0.95	32	0.12	0.02	0.96	0.046	-	-	-	-	-	-	-	-	-	-
AUDIT-C score																				
1–4 lower risk [Ref]	18					9					10					5				
5–12 hazardous/active AUD (f)	-	-	-	-	-	-	-	-	-	-	21	1.88	0.37	9.58	0.45	32	6.22	1.14	33.79	0.03
5–7 hazardous (m)	45	1.15	0.39	3.38	0.80	32	5.09	0.94	27.68	0.06	-	-	-	-	-	-	-	-	-	-
8–12 active AUD (m)	36	1.10	0.34	3.62	0.87	58	12.05	2.16	67.28	<0.01	-	-	-	-	-	-	-	-	-	-
Preferred bar type																				
Standard [Ref]	42					45					12					18				
Late	57	0.92	0.26	3.28	0.90	54	5.52	0.88	34.73	0.07	23	1.50	0.28	7.94	0.63	27	0.81	0.09	7.04	0.85
Occupation																				
Manager/professional											1	0.19	0.01	3.21	0.25	3	0.39	0.04	4.22	0.44
Technician/trade/labourer											8	0.26	0.05	1.45	0.12	7	0.09	0.01	0.55	<0.01
Community/personal service											7	0.54	0.12	2.42	0.42	8	0.41	0.09	1.96	0.27
Clerical/administrative/sales											6	0.12	0.02	0.58	<0.01	6	0.06	0.01	0.33	<0.01
Other [Ref]											9					13				
Pre-drinking																				
No	38	0.39	0.21	0.72	<0.01	39	0.53	0.28	1.00	0.05										
Yes [Ref]	61					60														
Typical night out?																				
No, usually smaller											11	4.22	1.17	15.28	0.03	10	2.44	0.61	9.76	0.21
No, usually bigger											5	0.64	0.17	2.35	0.50	7	0.57	0.15	2.13	0.40
Yes											15					20				
Session duration	99	1.22	1.04	1.42	0.01	99	1.46	1.25	1.70	<0.001	31	1.24	0.91	1.67	0.17	37	1.55	1.13	2.13	<0.01
Time																				
Before midnight [Ref]											18					18				
Midnight and after											19	2.57	0.81	8.16	0.11	13	4.62	1.39	15.32	0.01

Male model: Likelihood ratio χ^2^(14) = 62.1, *p <* 0.001; Female model: Likelihood ratio χ^2^(22) = 54.4, *p <* 0.001. f: Female. m: Male. n: Sample size. RR: Risk ratio. L/UCI: 95% Lower/upper confidence interval. [Ref]: Reference group. **^±^** age, energy drink use, and weekday were non-contributing variables in both models, occupation, whether it was a typical night out and time of survey were non-contributing variables in the male model, pre-drinking was a non-contributing variable in the female model. These non-contributing variables were removed in the backward stepwise selection approach.

## Data Availability

The data presented in this study are available on request from the corresponding author subject to ethical approval.

## References

[1] Stockwell T., Gruenewald P., Heather N., Stockwell T. (2004). Controls on physical availability of alcohol. The Essential Handbook of Treatment and Prevention of Alcohol Problems.

[2] Stockwell T., Chikritzhs T. (2009). Do relaxed trading hours for bars and clubs mean more relaxed drinking? A review of international research on the impacts of changes to permitted hours of drinking. Crime Prev. Commun. Saf..

[3] Popova S., Giesbrecht N., Bekmuradov D., Patra J. (2009). Hours and days of sale and density of alcohol outlets: Impacts on alcohol consumption and damage: A systematic review. Alcohol Alcohol..

[4] Hahn R.A., Kuzara J.L., Elder R., Brewer R., Chattopadhyay S., Fielding J., Naimi T.S., Toomey T., Middleton J.C., Lawrence B. (2010). Effectiveness of policies restricting hours of alcohol sales in preventing excessive alcohol consumption and related harms. Am. J. Prev. Med..

[5] Holmes J., Guo Y., Maheswaran R., Nicholls J., Meier P.S., Brennan A. (2014). The impact of spatial and temporal availability of alcohol on its consumption and related harms: A critical review in the context of UK licensing policies. Drug Alcohol Rev..

[6] Wilkinson C., Livingston M., Room R. (2016). Impacts of changes to trading hours of liquor licences on alcohol-related harm: A systematic review 2005–2015. Public Health Res. Pract..

[7] Sanchez-Ramirez D.C., Voaklander D. (2018). The impact of policies regulating alcohol trading hours and days on specific alcohol-related harms: A systematic review. Inj. Prev..

[8] Nepal S., Kypri K., Tekelab T., Hodder R.K., Attia J., Bagade T., Chikritzhs T., Miller P. (2020). Effects of Extensions and Restrictions in Alcohol Trading Hours on the Incidence of Assault and Unintentional Injury: Systematic Review. J. Stud. Alcohol Drugs.

[9] Sherk A., Stockwell T., Chikritzhs T., Andréasson S., Angus C., Gripenberg J., Holder H., Holmes J., Mäkelä P., Mills M. (2018). Alcohol Consumption and the Physical Availability of Take-Away Alcohol: Systematic Reviews and Meta-Analyses of the Days and Hours of Sale and Outlet Density. J. Stud. Alcohol Drugs.

[10] Jurkovich G.J., Rivara F.P., Gurney J.G., Fligner C., Ries R., Mueller B.A., Copass M. (1993). The Effect of Acute Alcohol Intoxication and Chronic Alcohol Abuse on Outcome from Trauma. JAMA.

[11] Hoonpongsimanont W., Ghanem G., Saadat S., Nguyen M., Louis C., Sahota P.K., Danishgar L., Carroll C., Barrios C., Lotfipour S. (2021). Correlation between Alcohol Use Disorders, Blood Alcohol Content, and Length of Stay in Trauma Patients. J. Emergencies Trauma Shock.

[12] Marcus J., Siedler T. (2015). Reducing binge drinking? The effect of a ban on late-night off-premise alcohol sales on alcohol-related hospital stays in Germany. J. Public Econ..

[13] Smith D.I. (1986). Comparison of patrons of hotels with early opening and standard hours. Int. J. Addict..

[14] Gilmore W., Symons M., Liang W., Graham K., Kypri K., Miller P., Chikritzhs T. (2021). Association between Nightlife Goers’ Likelihood of an Alcohol Use Disorder and Their Preferred Bar’s Closing Time: A Cross-Sectional Observational Study in Perth, Australia. Int. J. Environ. Res. Public Health.

[15] Coomber K., Zahnow R., Ferris J., Droste N., Mayshak R., Curtis A., Kypri K., de Andrade D., Grant K., Chikritzhs T. (2018). Short-term changes in nightlife attendance and patron intoxication following alcohol restrictions in Queensland, Australia. BMC Public Health.

[16] Graham K., Bernards S., Clapp J.D., Dumas T.M., Kelley-Baker T., Miller P.G., Wells S. (2014). Street intercept method: An innovative approach to recruiting young adult high-risk drinkers. Drug Alcohol Rev..

[17] Hughes K., Quigg Z., Bellis M.A., van Hasselt N., Calafat A., Kosir M., Juan M., Duch M., Voorham L. (2011). Drinking behaviours and blood alcohol concentration in four European drinking environments: A cross-sectional study. BMC Public Health.

[18] Miller P.G., Pennay A., Droste N., Jenkinson R., Quinn B., Chikritzhs T., Tomsen S.A., Wadds P., Jones S.C., Palmer D. (2013). Patron Offending and Intoxication in Night-Time Entertainment Districts.

[19] Miller P., Pennay A., Droste N., Butler E., Jenkinson R., Hyder S., Quinn B., Chikritzhs T., Tomsen S., Wadds P. (2014). A comparative study of blood alcohol concentrations in Australian night-time entertainment districts. Drug Alcohol Rev..

[20] Australian Institute of Health and Welfare (2020). National Drug Strategy Household Survey 2019.

[21] Reedy C., Gilmore W., Chikritzhs T. (2022). Estimated Alcohol-Attributable Deaths and Hospitalisations in Australia, 2010 to 2017. National Alcohol Indicators.

[22] MacKinnon D.P. (2011). Integrating Mediators and Moderators in Research Design. Res. Soc. Work. Pract..

[23] Miller P., Pennay A., Jenkinson R., Droste N., Chikritzhs T., Tomsen S., Wadds P., Jones S.C., Palmer D., Barrie L. (2013). Patron offending and intoxication in night-time entertainment districts (POINTED): A study protocol. A Study Protocol. Int. J. Alcohol Drug Res..

[24] Haber P. (2009). Guidelines for the Treatment of Alcohol Problems.

[25] Bowring A.L., Gouillou M., Hellard M., Dietze P. (2013). Comparing short versions of the AUDIT in a community-based survey of young people. BMC Public Health.

[26] Bush K., Kivlahan D.R., McDonell M.B., Fihn S.D., Bradley K.A. (1998). The AUDIT alcohol consumption questions (AUDIT-C): An effective brief screening test for problem drinking. Ambulatory Care Quality Improvement Project (ACQUIP). Alcohol Use Disorders Identification Test. Arch. Intern. Med..

[27] Dawson D.A., Grant B.F., Stinson F.S., Zhou Y. (2005). Effectiveness of the derived Alcohol Use Disorders Identification Test (AUDIT-C) in screening for alcohol use disorders and risk drinking in the US general population. Alcohol Clin. Exp. Res..

[28] Barry A.E., Chaney B.H., Stellefson M.L., Dodd V. (2015). Evaluating the psychometric properties of the AUDIT-C among college students. J. Subst. Use.

[29] Higgins-Biddle J.C., Babor T.F. (2018). A review of the Alcohol Use Disorders Identification Test (AUDIT), AUDIT-C, and USAUDIT for screening in the United States: Past issues and future directions. Am. J. Drug Alcohol Abus..

[30] Department of Local Government Sport and Cultural Industries Extended Trading Permits: Information on the Types of Extended Trading Permits. https://www.dlgsc.wa.gov.au/racing-gaming-and-liquor/liquor/liquor-licensing/extended-trading-permits.

[31] Independent Review Committee (2013). Liquor Control Act 1988: Report of the Independent Review Committee.

[32] Khadjesari Z., White I.R., McCambridge J., Marston L., Wallace P., Godfrey C., Murray E. (2017). Validation of the AUDIT-C in adults seeking help with their drinking online. Addict. Sci. Clin. Pract..

[33] Jones B.M. (1976). Male and female intoxication levels for three alcohol doses or do women really get higher than men?. Alcohol Tech Rep..

[34] Droste N., Miller P., Kaestle C.E., Curtis A., Hyder S., Coomber K., Pennay A., Chikritzhs T., Lam T., Gilmore W. (2018). Comparing levels of blood alcohol concentration and indicators of impairment in nightlife patrons. Drug Alcohol Rev..

[35] Australian Bureau of Statistics (2019). 1220.0—ANZSCO—Australian and New Zealand Standard Classification of Occupations, 2013, Version 1.3.

[36] IBM Corp (2020). IBM SPSS Statistics for Windows, Version 27.0.

[37] Gilmore W., Chikritzhs T., Stockwell T., Jernigan D., Naimi T., Gilmore I. (2016). Alcohol: Taking a population perspective. Nat. Rev. Gastroenterol. Hepatol..

